# Riparian Soil Pollution Caused by Sediment Metal Transport: Seasonal Changes and Ecological Risk Assessment

**DOI:** 10.3390/toxics12030213

**Published:** 2024-03-13

**Authors:** Ahmet Çelebi, Bülent Şengörür, Ali Torabi Haghighi, Ali Danandeh Mehr

**Affiliations:** 1 Department of Environmental Engineering, Faculty of Engineering, Sakarya University, 54050 Sakarya, Türkiye; 2 Faculty of Engineering, Kırklareli University, 39100 Kırklareli, Türkiye; 3 Water Energy and Environmental Engineering Research Unit, University of Oulu, 90014 Oulu, Finland; 4 Civil Engineering Department, Antalya Bilim University, 07190 Antalya, Türkiye; 5 MEU Research Unit, Middle East University, Amman 11831, Jordan

**Keywords:** pollution indexes, source identification, ecological risk, metal pollution, river health

## Abstract

The accumulation of pollutants in the sediment along surface water may negatively affect riparian zones and increase ecological risk. This article investigates the effects of metal sediments on riparian soil via field monitoring and ICP-OES analysis. To this end, pollution levels, seasonal changes, and potential sources of the pollutants were determined for the Melen River watershed, Turkey. The ecological statuses (contamination factor, enrichment factor, index of geo-accumulation, pollution index, modified pollution index, and potential and modified ecological risk indexes) of the watershed were also analyzed. Although no significant seasonal differences in the metal sediments were observed, their spatial distribution in the sediments and riparian soils varied markedly. Cr (11.4 to 136), Co (7.7 to 21.52), Cu (11.4 to 76.6), and Ni (14.06 to 128.2) recorded as mg/kg significantly increased from the upstream to the downstream. The metals possessing the highest risk in the sediment and riparian soil regarding the river health were Cu, Co, and Ni. The risk values were found to be heavily polluted (*PI* > 3 and *MPI* > 10), and the risk indexes were above the “desired environment without the risk”. The risk index was found to be more than 50, and the modified risk indexes exceeded 200 at many points. The transportation of pollutants in surface water became evident in the sediment, resulting in adverse effects on the riparian zone and the ecological system.

## 1. Introduction

River ecosystems are the main vessels that feed the earth and life. The composition of rivers, along with their pollution levels, plays a crucial role in shaping ecosystem dynamics. Among the various pollutants, metals stand out as significant contributors, demanding careful consideration due to their toxic and carcinogenic potential. Understanding their presence and impact is vital for deciphering the intricate mechanisms within ecosystems [1,2,3,4]. Although certain metals serve as essential building blocks for aquatic life, their concentration beyond specific threshold values can lead to detrimental effects on living organisms [5,6]. Metal persistence and accumulation in an aquatic environment can pose a long-term danger to its ecosystem [7]. In river ecosystems, metals have the potential to accumulate in sediment or undergo transport over hundreds of kilometers in suspended and dissolved forms [8,9,10,11].

The source of metals in aquatic environments can be geogenic or anthropogenic. Pollution can occur naturally by weathering or artificially by human activities [12,13,14]. With the increase in human population, river ecosystems are exposed to the greatest threat. Riparian zones are particular transition areas between aquatic and terrestrial environments and are sensitive to the level of metals [15]. They perform essential roles such as nutrient filtration, flood mitigation, erosion reduction, water purification, and flow regulation [16,17,18]. Seasonal flow differences in rivers can significantly affect the riparian soil and sediment. Regarding environmental health, metals reaching the riparian zone should be monitored, and the associated risk needs to be assessed [19,20].

Various approaches have been developed to detect primary pollutants in river ecosystems’ sediments and soils and determine their risks [21,22,23]. The geoaccumulation index (*Igeo*), enrichment factor (*EF*), and contamination factor (*CF*) were the commonly applied methods. In addition, the pollution index (*PI*) and modified indexes have also been used. Ecological risk indexes and their modified methods are useful as risk assessment methods for the health of the aquatic ecosystem [7,24].

While studies exist to assess the pollution levels and sources of sediments, comprehensive examinations that encompass both sediment and soil within river ecosystems are scarce. Furthermore, only a limited number of studies have delved into the seasonal risk assessments of pollutants in the sediment and soil of river ecosystems. Moreover, the extent to which riparian soil impacts aquatic ecosystems and the associated risk dimensions have not been thoroughly investigated in the context of environmental health. The primary objectives of this study are threefold: (i) to elucidate the transport dynamics of metals along the river and the interactions between riparian soil and sediment; (ii) to identify and assess metal pollution and its sources in both sediment and riparian soil; and (iii) to conduct a comprehensive investigation into the seasonal variations of sediment and riparian soil pollution, pollution sources, and ecological risk assessments. Notably, this research represents the first attempt to explore these aspects within the Melen River watershed in Turkey.

## 2. Materials and Methods

### 2.1. Study Area

**Figure 1:**
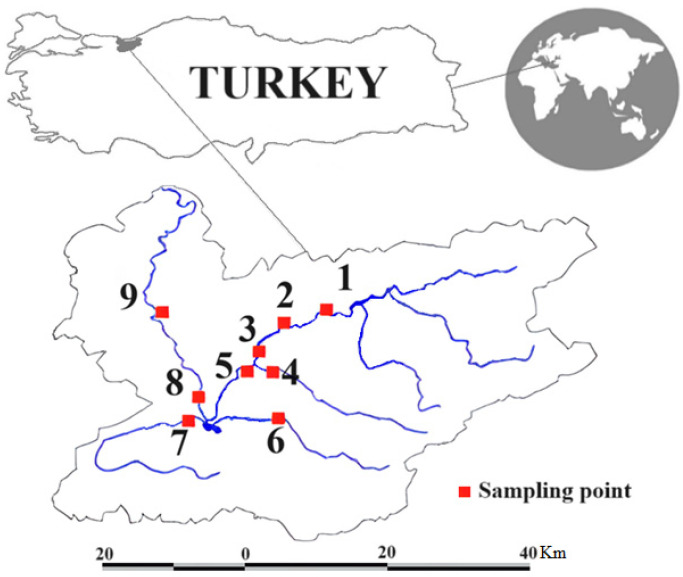
The Melen River watershed and the location of nine sampling points.

The Melen Rivers watershed in Turkey was chosen as the study area (Figure 1). It is located between 40° and 42° north latitude, and 30° and 33° east longitude. The Melen River pours into the Black Sea. The land use of the basin is 40% forest (natural meadows, coniferous forests), 32.5% agricultural lands, 3.5% meadows and pastures, and 24% settlements and other areas [25].

The mean annual temperature of the basin is 13.5 °C, the annual precipitation is 822.8 mm, and the average relative humidity is 75.4%. The lowest temperatures occur between December and March, and the highest between May and August. Throughout the year, 21% spring, 14% summer, 19% autumn, and 46% winter precipitation are seen. The average temperature 19–20 °C in September, with an average of 4–5 °C in November–December. It drops to an average of 3–4° C in January as the coldest month. After mid-February, the average temperature usually rises. Wind speed may be interrupted due to the mountains surrounding the study area. Due to the low wind speed in autumn and winter, foggy weather events are seen intensely (36.1 days/year). While evaporation may decrease to 0 mm in the winter months (December, January, February, March), it increases from April to July, and the highest evaporation rate is observed in July. The evaporation decreases again between August and November and reaches the lowest rate in November [26].

The average population of the basin consists of four hundred thousand and includes three active organized industrial zones. The main sectors are the automotive, textile, food processing, and forest product industries. Many polluting factors such as agricultural chemical fertilizers, irregular use of meadows and pastures, livestock practices, domestic or industrial wastes originating from settlements, pollution caused by vehicles on highways, and mining activities pose pollution risk for the study area. The percentage of total organic carbon in the basin’s sediment is between 0.23 and 3.30 [27,28].

### 2.2. Sampling

Sediment and riparian soil samples were collected from nine points four times in autumn (November), winter (February), spring (May), and summer (August). The samples were collected using a 0.1 m^2^ Van Veen sediment trap. Each sample was taken with stainless steel grub from the top layer of the sediment surface (0–5 cm) and transported to a glass jar (250 mL). The samples were placed in an ice cooler before being transferred for storage in the laboratory and then transported with a cooling system. Surface samples can be taken to monitor the change and accumulation of pollutants in sediment and soil throughout the year. Although seasons cannot be clearly separated in different regions, annual pollution can be revealed by surface sampling [29,30].

Each riparian soil sample was collected as a composition of five sub-samples. Triplicate samples were created from each point. River riparian soil samples were collected from the surface (0–20 cm depth). The leaves and non-soil materials were removed. In an area of 1 m^2^, the five samples were mixed in equal amounts. All soil samples were stored in sealed polyethylene bags and then kept at 4 °C in the refrigerator for further analysis. Approximately 250 g of soil was collected from each point and was packaged and stored at room temperature [20,29]. The coordinates of the sampling points (riparian soil and sediment) are tabulated in Table 1.

### 2.3. Sample Preparation, Analytical Instruments, and QA/QC

The sediment samples were dried with the help of Teknosem brand lyophilizer for one day. The dried samples were ground and homogenized. The method used it as an analytical procedure for twelve metals (US EPA M.1613). The analysis of metals (Al, Ba, Co, Cr, Cu, Fe, P, Mn, Ni, Pb, V, and Zn) was measured in the surface sediment and riparian soil. Cleaning processes were carried out. Sample cups were cleaned with 1:1 diluted hot HCl and HNO_3_ for at least 2 h and dried after washing with distilled water. Polymeric or glassware storage containers were washed with dilute acid solutions.

Of the surface soil and sediment samples, 0.1 g was weighed in a microwave Teflon cup, then 4 mL of concentrated HNO_3_, 2 mL of concentrated HCl, and 1 mL of concentrated HF were added. Digestion was carried out at 180 °C under high pressure for 20 min. About 300 mg of solid H_3_BO_3_ was added to the chilled samples to neutralize. The acidic extracts in a Teflon container were diluted in 50 mL bottles. These samples were filtered and stored in polyethylene bottles until analysis in the ICP-OES instrument [30]. Autotuning was performed for optimum sensitivity conditions. All sampling equipment was thoroughly washed and rinsed with acetone, followed by hexane, before sampling to avoid cross-contamination and reveal background contamination. Solvent rinses were analyzed as control samples and applied the same extraction procedure as the soil and sediment samples from the river. In the blank samples, no significant target analyses were detected [31,32]. Quality control was prepared for the calibration by using a 1000 mg L^−1^ standard solution from Merck (multi-element standard solution IV). The limits of the detection values for Al, Co, Cr, Cu, Fe, Mn, Ni, Pb, V, and Zn were 0.63, 0.06,0.23, 0.11, 0.74, 0.08, 0.13, 0.30, 0.05, and 0.069 mg/kg, respectively. The limits of the quantitation values were 1.93, 0.18, 0.78, 0.35, 2.46, 0.25, 0.41, 0.97, 0.16, and 0.25 mg/kg, respectively. Calibration curves (r^2^) (ranging from 0.9956 to 0.9998) were generated automatically for each metal. Three replicates were used for the measuring.

### 2.4. Statistical Analysis

Statistical analysis of the compositional metal concentrations in the river riparian soil and surface sediment samples was performed using Microsoft Excel, CoDAPack version 2.03.01, and SPSS version 25.0. Principal component analysis (PCA) was applied to identify the possible metal sources measured in riparian soil and sediment samples [6]. Extraction was performed during PCA analysis. Raw calculated factor loading coefficients varimax-rotated with Kaiser normalization. Only the leading principal components with significance were considered.

Normality tests (Kolmogorov–Smirnov and Shapiro–Wilk) were applied to all data. Skewness and kurtosis values were evaluated according to the normal distribution. As a result of these analyses, it was observed that some parameters had higher skewness and curvature and therefore did not show normal distribution.

Composition data requiring transformation has been made available to make statistical results more accurate. Standard statistical approaches of principal component analysis (PCA) were applied. Such methodologies were proposed in the early 1980s for the first time [33]. Components only give information about their relative sizes. A data analysis transformation was conducted focusing on the components and the ratios between components [34]. Log-ratio transformation (clr) by the CoDAPack code to “open” the raw data is expressed as follows:

clr(x) = (log(x1/g(x)), …, log(xD/g(x)))

where x represents the composition vector, g(x) is the geometric mean of the x composition, and xD is the Euclidean distance between the individual residue variables. Clr conversions were performed using CoDaPack Edition 2.03.01, which is an Excel-based software for combinatorial data transformation [35]. The normality of the transformed data was tested. Skewness and kurtosis were observed normally. The correlation between parameters was observed. As a result of PCA analysis, the elements were divided into groups and biplot shapes were created.

### 2.5. Risk Assessment and Toxicity Analysis

The contamination and ecological risks of metals in the riparian soil and sediment were calculated. Environmental factors and indexes such as the contamination factor (*CF*), enrichment factor (EF), index of geo-accumulation (*Igeo*), modified and standard pollution index (*PI* and *MPI*), and potential and modified ecological risk indexes (*RI* and *MRI*) were analyzed.

*CF* is a single and straightforward index indicator used to assess metal contamination. The element in the sampling area and the same element in the background proportion provide a reference value (Equation (1)) [7].

(1) $$CF=\frac{C_{i}}{C_{b}},$$

where *CF* is the contamination factor, *C_i_* is the metal concentration in a region, and *C_b_* is the concentration of the same metal in the background or reference. Ef is an enrichment factor as a useful single-element index that assumes no anthropogenic input and little or no weathering (Equation (2)).

(2) $$EF=\frac{{\left(c_{i}/c_{\mathrm{ref}\;}\right)}_{\mathrm{sample}\;}}{{\left(c_{i}/c_{\mathrm{ref}\;}\right)}_{\mathrm{background}\;}}$$

*Igeo* is also a single-element index that describes metal contamination in sediments or soil by comparing the current levels with the before levels, which is calculated using Equation (3).

(3) $$Igeo=\log_{2}\left(\frac{C_{i}}{1.5\times B_{i}}\right)$$

where *C_i_* is the measured metal concentration in riparian soil and the sediment, and *B_i_* is the metal’s geochemical background concentration or reference value. The 1.5 factor accounts for anthropogenic effects and possible background value variations [6].

The modified and standard pollution indexed (*MPI* and *PI*), which are improvements in the pollution index, use *CF* and *EF* in their calculation (Equations (4) and (5)) [22].

(4) $$PI=\sqrt{\frac{{\left(Cf_{\mathrm{average}\;}\right)}^{2}+{\left(Cf_{\max}\right)}^{2}}{2}}$$

(5) $$MPI=\sqrt{\frac{{\left(Ef_{\mathrm{average}\;}\right)}^{2}+{\left(Ef_{\max\;}\right)}^{2}}{2}}$$

The *RI* and *MRI* also measure the susceptibility of the biological community to generally contaminate the river [21]. They consider the *CF* and *EF* of the elements, potential ecological risk factors (*Er*), and sediment logical toxic response factors (*Tr^i^*) (Equations (6) and (7)).

(6) $$RI=\overset{n}{\underset{i=1}{\sum }}Er^{i}=\overset{n}{\underset{i=1}{\sum }}Tr^{i}\times CF^{i}$$

(7) $$MRI=\overset{n}{\underset{i=1}{\sum }}Er^{i}=\overset{n}{\underset{i=1}{\sum }}Tr^{i}\times EF^{i}$$

## 3. Results

### 3.1. Riparian Soil and Sediment Pollutant Levels

Riparian soil metal averages were found to be higher than sediment (except Ni). The averages for all metals in riparian soil and sediment are close in the basin. Although riparian soil had a higher average, the highest values for all metals (except Pb and P) were found in sediments. Metals in the watershed were measured and detected at all sample points. The level of each metal in the surface sediments and riparian soil is presented in supplementary materials (see Table S1).

**Figure 2:**
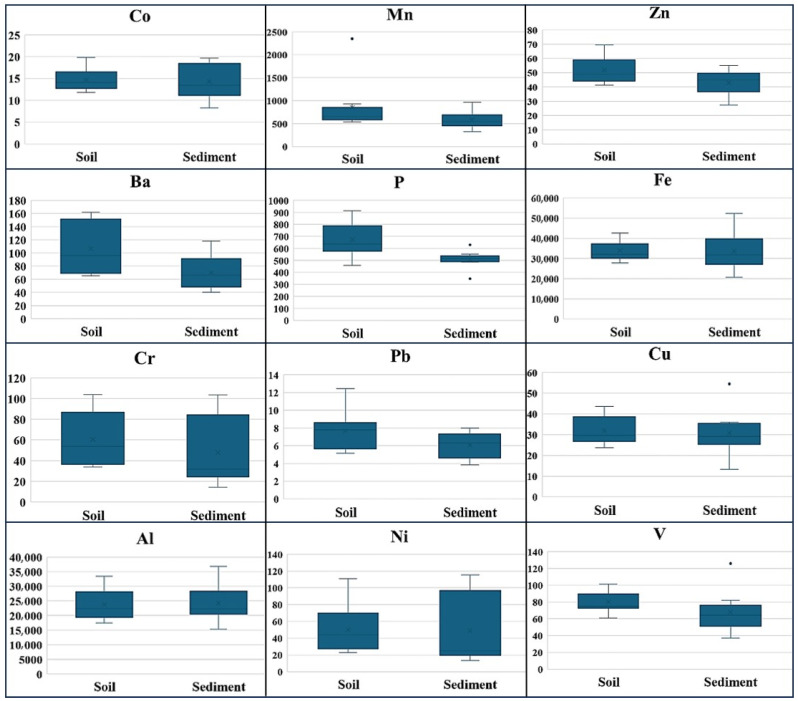
Mean riparian soil and sediment concentrations (mg/kg dry matter) in the Melen watershed.

In the river system, Cr was found to be the lowest at 11.4 and the highest at 136 mg/kg. The lowest chromium values were observed at point 3 and the highest values at point 9. At point number 4, an increase in accumulation stood out. Cobalt concentration was observed in the range of 5 to 20 mg/kg, and points 2, 8, and 9 were the highest valued points. The lowest value was observed at point 3. Copper was in the range of 10–50 mg/kg. The lowest values were observed at point 3 and the highest at point 6. Lead was in the range of 4 to 10 mg/kg. The lowest values were observed at sample point 3. Nickel was in the range of 20–150 mg/kg, and the highest value was observed at point 7. Vanadium was in the range of 35–140 mg/kg, and the highest value was observed at point number 6 and point 3 again had the lowest value. The lowest value was observed at point 3 again for zinc, which was in the range of 25–75 mg/kg. The lowest values of Al were seen at point 3, and the highest values were seen at sample point 6. It was in the range of 15,000 to 45,000 mg/kg. A similar situation was found for Fe. The lowest value was observed at point 3 and the highest value at point 6 in the range of 18,000–60,000 mg/kg. Manganese was observed at point 1 at the highest and point 5 at lowest, in the range of 250 to 620 mg/kg. Barium was found in the range of 40 to 230 mg/kg, with the lowest values at points 3, 6, and 7 (Figure 2).

The seasonal pollution levels were observed close to each other regarding all metals in the sediment and riparian soil. Seasonally, the highest average Ba (80.3–106.3), Cr (57.3–60.5), Mn (636–619), Ni (605–673), P (605–673), and V (78.2–80.2) values (mg/kg dry matter) in the watershed sediments and soils were observed in autumn. The highest values for any metal were not observed in the summer period. On the contrary, the lowest Ba, (61.3–88.3) Cr (42.5–44.1), Cu (27.9–29.7), Fe (32,056–32,659), Ni (46.7–45.8), V (59.1–61.8), and Z (40.8–49.6) values (mg/kg dry matter) were observed in the summer period. Generally, the seasonal variation in pollutants was around 10%, and the highest intra-annual variation of about 30% was observed in V and Cr. The seasonal average of pollutants in the study area is shown in Tables S1 and S2. The values were found to be close to the average of other studies (except Pb). Ni values were found to be slightly higher in the Melen River system sediment and riparian soil, and the Pb value was relatively low (Table 2).

With the effect of precipitation and other factors, the water level in the riparian zone area was dynamic. Ecological processes and metal exchange in sediments and riparian soils are overly complex [18,42,43].

### 3.2. Interaction of the Pollutants

Certain metals showed a significant correlation, but some did not show a significant relationship in terms of sediment and riparian soil metals. Significant positive correlations were observed between metals in riparian soil and sediment (Tables S3 and S4). Correlation results indicated the presence of pollutant groups. Correlations among the pollutants reflect their origin [36]. If no significant correlation was observed among the elements, the contaminants were not sourced by a single factor [1].

Although the metal pollution levels did not differ significantly in the river system seasonally, significant differences were found between the spatial values. The significant values of the ANOVA test to measure the seasonal and spatial variation of each metal measured are shown in Tables S5 and S6. According to the ANOVA test results, only P and Pb differed seasonally. It was found that other metals did not vary in sediment and soil throughout the year. Spatially, all metals differed in the sediments and soils of the study area. In another study on sediment seasonal differences, Fikirdeşici et al. [44] did not find statistical significance among the results during the year. Some seasonal changes were observed for Ni, Mn, and Al. The seasons with the most remarkable difference were between spring and summer. The highest seasonal differences were observed in our study for Ni, Cr, Mn, and Al metals.

In the statistical analysis carried out to determine the source of the pollutants, it was observed that these metals reached the drinking water source and accumulated from three different source sediment and riparian soil. The principal component analysis results for all pollutant groups are combined in Tables S7 and S8. PCA results showed that for metals of riparian soil, the first three major components (Eigenvalue > 1) accounted for 84.37% of the total variability between samples (39.15% for PC1, 31.40% for PC2, 13.81% for PC3). PC2 included Ba, Cr, Ni, and P, and PC3 included Pb and Al congeners. Therefore, most of the metal variation in the dataset can be explained by the first three components in the riparian soil of the Melen watershed. Three primary metal pollutants were found in riparian soil and sediment in the basin. The primary metal pollutant sources for the basin are industry, agricultural activities, and weathering [26]. These activities are the main polluting factors in riparian soil and sediment. Industry stands out as the first component (PC1) containing copper, iron, cobalt, manganese, vanadium, and zinc. The second factor (PC2) represents agricultural activities, and the third component (PC3) represents pollution caused by weathering.

**Figure 3:**
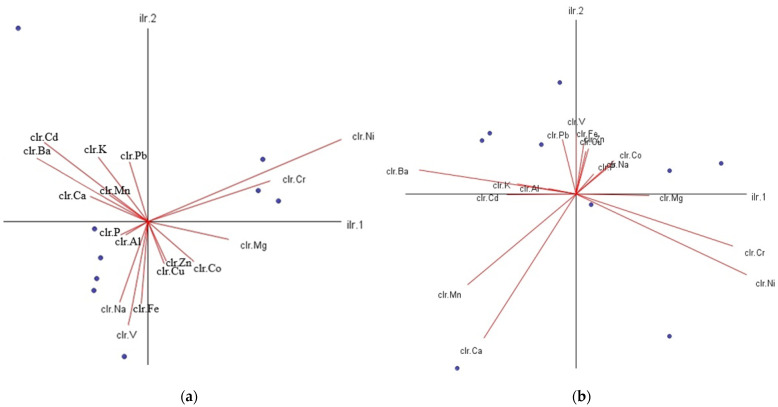
Metal scattering in the Melen River: (**a**) sediment; and (**b**) riparian soil.

Regarding the metals in sediment, the first three components (Eigenvalue > 1) accounted for 78.20% of the total variability between the samples (36.60% for PC1, 32.73% for PC2, 22.65% for PC3). PC2 included Ba, Cr, Ni, and Al, and PC3 only the Pb congener (Tables S7 and S8). Therefore, most of the metal variation in the dataset can be explained by the first three components in the sediment of the study area. In both sediment and riparian soil, Co, Cu, Fe, Mn, V, and Zn clustered in the first group, Ni, Cr, and Ba in the second, and P in the third group (Figure 3).

### 3.3. Risk Assessments of the Pollutants

Severe pollution and risk were found in basin riparian soil and sediment in terms of many metals (Tables S9–S11). Although low metal values were observed individually in the river system, “significant contamination” was detected as a contamination factor, “significant enrichment” of some metals as an enrichment factor, and “moderate contamination” in terms of the *Igeo*. “Undesired ecological risk” was found for many metals and points for the risk index calculations. All risk assessments (*CF*, *EF*, *Igeo*, *RI*, *MRI*, *PI*, and *MPI*) are combined in Table S9 for sediment and Table S10 for riparian soil. The associated classes are shown in Table 3 and Table 4.

The *EF* and *CF* of the sediments and riparian soil were analyzed for the metals used to assess the contamination. Four *CF* categories suggested by [21] are described as *CF* < 1 low contamination; 1 ≤ *CF* < 3, moderate contamination; 3 ≤ *CF* < 6, significant contamination; and *CF* ≥ 6 very high contamination. However, *EF* has seven categories: *EF* < 1 no enrichment; 1–3 minor; 3–5 moderate; 5–10 significant; 10–25 high; 25–50 very high; and *EF* > 50 extremely high enrichment.

Most of the metals in the sediment were found to be uncontaminated, but Ni reached the highest *CF* values at points 7 and 9 (5.32 and 4.8). Very severe contamination was not observed at any sample point. Mn and Ni are significantly contaminated at some points (1, 7, and 9) of riparian soil. All other metals were observed as uncontaminated or moderately contaminated. Very high and extremely high enrichment was not observed for any pollutant in sediment and riparian soil samples. The highest *EF* values were reached in Ni, Cu, and Co in the sediment, and Mn and Ni in the soil samples. The lowest values were observed for Cr, V, and Zn (Table 3).

The *Igeo* classification is like *EF*. But for Igeo, if < 0 uncontaminated, 0–1 noncontaminated, 1–2 moderately contaminated, 2–3 slightly to heavily, 3–4 heavily, 4–5 heavily to extremely, >5 extremely contaminated. Uncontaminated values for metals were observed in most of the sample points. Moderate contamination was found in the sediment only for nickel at sample points 8 and 9. Heavily, heavily to extremely, and extremely contaminated values were not observed. Only the contamination values for Ni were found contaminated in riparian soil. Uncontaminated values were observed for all other metals in terms of *Igeo*. The highest values were again found at sample points 7, 8, and 9 (Table 3).

The *PI* and modified pollution index (*MPI*) analyses were made to measure the ecological situation and risk levels. *MPI* is a sensitive index that uses an enrichment factor, classified as follows: unpolluted <0.7 for *PI*, the value < 1 for *MPI* indicates unpolluted; *PI* value > 3 or *MPI* value > 10 is classified as heavily polluted [7]. According to *PI*, the sediment samples were only heavily polluted by Ni. The same situation was found according to *MPI*. Riparian soil samples were heavily polluted by Mn and Ni. The same situation was found for *MPI* [30].

The *RI* was created for a large-scale ecological risk analysis. If there is an *RI* or *MRI* value less than 150, it indicates the desired environment without ecological risk, while if the *RI* or *MRI* value exceeds 600, it means very high risk. In most sample points, the ecological risk was not observed in the sediment and riparian soil regarding *RI* and *MRI*. The highest risk areas for sediment were at sample points 7 and 8, and the riskiest areas for soil were at sample points 6 and 7. High ecological risk was not observed at any point (Table 2 and Table 3).

Because the *EF* values were higher in all areas other than *CF*, the *MRI* values were found to be high at the points. As a result of the high Ni and Mn values in the study area, the highest ecological risk factor is Ni for the riparian soil and sediments. Slight differences were observed between the results of ecological factors and risk assessments seasonally (Table S9). While the highest risk factors were found in summer, the lowest risks and contamination occurred in autumn.

In the Liaohe River (China), riparian soil and sediment were monitored, and risk assessments were made [20]. Similar to our study, the riparian zone soil risk values were higher for heavy metals such as Cr, Ni, Zn, and Cu than in the sediment. The results indicate more accessible transport for these metals in the sediment.

As a result of the pollution of water resources, an extra pollution situation occurred in the riparian soil, especially in downstream areas. They have essential roles for the riparian zone and soil ecosystem health. To monitor and evaluate river and ecosystem health, riparian soil should be considered an essential factor. Rivers, wetland, and their coastal areas, such as riparian forests, are closely interrelated [45,46]. For riparian and buffer zones, pollution retentions have been inadequately evaluated to date [47,48]. Riparian soil should be given importance for ecosystem and river health, and their risk assessments should be made.

## 4. Conclusions

Pollution transport in surface water and its accumulation in sediment adversely affect the riparian soil and ecosystem health. While many recent studies have focused on soil pollution detection and modeling (e.g., [49,50,51]), the importance of the interconnection between soil pollution and river ecosystems was underscored in our study. It is necessary to consider the sediment and riparian soil together, to make ecological risk assessments, and assess ecosystem health. Risky metals were carried by the sediment along the river and reached levels that cause toxic effects downstream. Metal pollution caused by industrial facilities, agricultural activities, and natural erosion threatens the river ecosystem. No significant seasonal differences were observed for sediment and riparian soil, but spatially, almost all points were found to be statistically different from each other. The ecological risk values differed with the pollution of the riparian soil from the sediment at many points. Even if the individual pollutants appear below specific standards, the health of river ecosystems can be maintained with holistic ecological risk assessments.

Our study focused on assessing the metal content in riverbank sediment along the Melen River through field measurements. Future research endeavors could integrate remote sensing and emerging satellite-based data to evaluate metal pollution in the sediment and riparian soil of tributaries that pose challenges for direct field measurement due to limited accessibility. Moreover, the adjustment of the number of monitoring stations and evaluated pollution parameters should be considered based on the size of the basin under investigation. Future investigations should also explore the toxic effects induced by various pollutants, particularly those impacting both agricultural ecosystems and human health [52,53]. Our results indicated that the accumulation of pollutants in the downstream areas of the basins leads to elevated levels of riparian soil pollution and ecological risks. Consequently, this emphasizes the crucial role of riparian areas in environmental risk assessment and underscores the need for increased attention to their management.

## Figures and Tables

**Table 1:** Sampling point coordinates.

Point	N	E	Elevation (m)	Location
1	40.907455	31.22219	225	upstream
2	40.887861	31.16137	202	upstream
3	40.843652	31.13385	166	upstream
4	40.835489	31.11113	174	tributary
5	40.836464	31.10105	164	urban area
6	40.770528	31.10696	168	tributary
7	40.763547	30.99814	155	tributary
8	40.822176	31.02494	151	downstream
9	40.864534	30.98388	171	downstream

**Table 2:** Concentration of metals in the river surface sediments and riparian zone soils (mg/kg).

Type	Location	Cr	Ni	Zn	Pb	Cu	References
Sediment	Liaohe River, China	38	27	37	17	28	[20]
	Yangtze River, China	89	37	174	60	82	[36]
	Louro River, Spain	108	46.4	-	61.8	45.4	[37]
	Swarnamukhi River, India	85	2	63	21	100	[38]
	Linggi River, Malaysia	33	10	102	30	14	[39]
	Gorges River, Australia	39	13	157	67	30	[40]
	Red River, Vietnam	85	38	127	66	83	[41]
	Melen River, Turkey	57.27	50.9	42	5.15	29.3	This study
Riparian soil	Liaohe River, China	34	23	31	17	25	[20]
	Beiyun River, China	22.9–114.6	8.8–45.6	36.1–478.3	11.6–61.8	7.1–125.7	[18]
	Red River, Vietnam	81	37	127	68	98	[41]
	Melen River, Turkey	60.53	49.9	51.9	7.71	31.97	This study

**Table 3:** Sediment risk assessment classification.

					Point							
		1	2	3	4	5	6	7	8	9	*PI*	*MPI*
*CF*	Cr	**□**	**□**	**□**	**□**	**□**	□	**●**	**□**	**□**	**□**	**●**
	Co	**□**	**□**	**□**	**□**	**□**	**●**	**●**	**□**	**●**	**●**	**●**
	Cu	**□**	**□**	**□**	**□**	**●**	**●**	**●**	**●**	**●**	**●**	**●**
	Pb	**□**	**□**	**□**	**□**	**□**	**□**	**□**	**□**	**□**	**□**	**●**
	Ni	**●**	**□**	**□**	**●**	**□**	**●**	**+**	**+**	**+**	**●**	**+**
	V	**□**	**□**	**□**	**□**	**□**	**●**	**□**	**□**	**□**	**●**	**●**
	Zn	**□**	**□**	**□**	**□**	**□**	**□**	**□**	**□**	**□**	**□**	**●**
	Al	**□**	**□**	**□**	**□**	**□**	**□**	**□**	**□**	**□**	**□**	**●**
*EF*	Cr	**●**	**●**	**●**	**●**	**●**	**●**	**×**	**+**	**+**		
	Co	**●**	**+**	**+**	**+**	**+**	**●**	**×**	**+**	**+**		
	Cu	**+**	**+**	**+**	**+**	**×**	**×**	**×**	**×**	**×**		
	Pb	**●**	**●**	**●**	**●**	**●**	□	**●**	**●**	**●**		
	Ni	**+**	**+**	**+**	**×**	**+**	**+**	■	■	■		
	V	**●**	**●**	**●**	**+**	**+**	**+**	**+**	**+**	**●**		
	Zn	**●**	**●**	**●**	**●**	**+**	**●**	**+**	**+**	**+**		
*Igeo*	Cr	**□**	**□**	**□**	**□**	**□**	**□**	**□**	**□**	**□**		
	Co	**□**	**□**	**□**	**□**	**□**	**□**	**□**	**□**	**□**		
	Cu	**□**	**□**	**□**	**□**	**□**	**●**	**□**	**□**	**□**		
	Pb	**□**	**□**	**□**	**□**	**□**	**□**	**□**	**□**	**□**		
	Ni	**□**	**□**	**□**	**□**	**□**	**□**	**+**	**+**	**+**		
	V	**□**	**□**	**□**	**□**	**□**	**□**	**□**	**□**	**□**		
	Zn	**□**	**□**	**□**	**□**	**□**	**□**	**□**	**□**	**□**		
	Al	**□**	**□**	**□**	**□**	**□**	**□**	**□**	**□**	**□**		
*RI*	□	**□**	**□**	**□**	**□**	**□**	**□**	**□**	**□**	**□**		
*MRI*	□	**□**	**□**	**□**	**□**	**□**	**□**	**●**	**●**	**●**		

*CF*: <1 low contamination: **□** 1–3 moderate cont.: **●** 3–6 significant cont.: **+**. *EF*: <1 no enrichment: **□** 1–3: minor: **●** 3–5: moderate: **+** 5–10: significant: **×** 10–25: high enrichment: ■. *Igeo*: <0 uncontaminated: **□** 0–1 noncontaminated: **●** 1–2 moderately: **+** 2–3 slightly to heavily. *PI*: <0.7 unpolluted: **□** 0.7–3 polluted: **●** >3 heavily polluted: **+**. *MPI*: <1 unpolluted: **□** 1–10: polluted: **●** >10 heavily polluted: **+**. *RI* and *MRI*: <150: no ecological risk: **□** 150–300 moderate: **●** 300–600 high: **+**.

**Table 4:** Soil risk assessment classification.

Point												
		1	2	3	4	5	6	7	8	9	*PI*	*MPI*
*CF*	Al	**□**	**□**	**□**	**□**	**□**	**□**	**□**	**□**	**□**	**□**	**□**
	Co	**□**	**□**	**□**	**□**	**□**	**□**	**●**	**□**	**●**	**●**	**●**
	Cr	**□**	**□**	**□**	**□**	**□**	**●**	**●**	**□**	**□**	**●**	**●**
	Cu	**●**	**□**	**□**	**●**	**●**	**●**	**●**	**□**	**●**	**●**	**●**
	Fe	**□**	**□**	**□**	**□**	**□**	**□**	**□**	**□**	**□**	**□**	**●**
	Mn	**+**	**●**	**●**	**●**	**●**	**●**	**●**	**●**	**●**	**●**	**+**
	Ni	**●**	**●**	**●**	**●**	**●**	**●**	**+**	**●**	**+**	**●**	**+**
	Pb	**□**	**□**	**□**	**□**	**□**	**□**	**□**	**□**	**□**	**□**	**●**
	V	**□**	**□**	**□**	**□**	**□**	**□**	**●**	**□**	**□**	**●**	**●**
	Zn	**□**	**□**	**□**	**□**	**□**	**□**	**●**	**□**	**□**	**●**	**●**
*EF*	Co	**●**	**+**	**+**	**+**	**+**	**+**	**+**	**+**	**+**		
	Cr	**●**	**●**	**●**	**●**	**●**	**×**	**+**	**+**	**●**		
	Cu	**+**	**+**	**+**	**×**	**×**	**×**	**×**	**+**	**+**		
	Fe	**●**	**●**	**+**	**+**	**●**	**+**	**+**	**●**	**●**		
	Mn	**■**	**×**	**×**	**×**	**×**	**×**	**×**	**×**	**×**		
	Ni	**×**	**×**	**×**	**×**	**+**	**■**	**■**	**×**	**■**		
	Pb	**●**	**●**	**+**	**●**	**●**	**●**	**●**	**●**	**●**		
	V	**●**	**+**	**+**	**+**	**+**	**+**	**+**	**+**	**●**		
	Zn	**●**	**+**	**+**	**+**	**+**	**+**	**+**	**+**	**+**		
*Igeo*	Al	**□**	**□**	**□**	**□**	**□**	**□**	**□**	**□**	**□**		
	Co	**□**	**□**	**□**	**□**	**□**	**□**	**□**	**□**	**□**		
	Cr	**□**	**□**	**□**	**□**	**□**	**□**	**□**	**□**	**□**		
	Cu	**□**	**□**	**□**	**□**	**□**	**□**	**●**	**□**	**□**		
	Fe	**□**	**□**	**□**	**□**	**□**	**□**	**□**	**□**	**□**		
	Mn	**+**	**□**	**□**	**□**	**●**	**□**	**●**	**□**	**●**		
	Ni	**●**	**□**	**□**	**□**	**□**	**+**	**+**	**●**	**+**		
	Pb	**□**	**□**	**□**	**□**	**□**	**□**	**□**	**□**	**□**		
	V	**□**	**□**	**□**	**□**	**□**	**□**	**□**	**□**	**□**		
	Zn	**□**	**□**	**□**	**□**	**□**	**□**	**□**	**□**	**□**		
*RI*		**□**	**□**	**□**	**□**	**□**	**□**	**□**	**□**	**□**		
*MRI*		**□**	**□**	**□**	**□**	**□**	**●**	**●**	**□**	**●**		

*CF*: <1 low contamination: **□** 1–3 moderate cont.: **●** 3–6 significant cont.: **+**. *EF*: <1 no enrichment: **□** 1–3: minor: **●** 3–5: moderate: **+** 5–10: significant: **×** 10–25: high enrichment: **■**. *Igeo*: <0 uncontaminated: **□** 0–1 noncontaminated: **●** 1–2 moderately: **+** 2–3 slightly to heavily. *PI*: <0.7 unpolluted: **□** 0.7–3 polluted: **●** >3 heavily polluted: **+**. *MPI*: <1 unpolluted: **□** 1–10: polluted: **●** >10 heavily polluted: **+**. *RI* and *MRI*: <150: no ecological risk: **□** 150–300 moderate: **●** 300–600 high: **+**.

## Data Availability

The datasets used and/or analyzed during the current study are available from the corresponding author on reasonable request.

## Supplementary Materials

The following supporting information can be downloaded at: https://www.mdpi.com/article/10.3390/toxics12030213/s1, Table S1: Seasonal and spatial average metal concentration (mg/kg) of the sediment; Table S2: Seasonal and spatial average metal concentration (mg/kg) of the soil; Table S3: Sediment metal correlation in the watershed; Table S4: Riparian soil metal correlation in the watershed; Table S5: Seasonal ANOVA test results of the watershed metals; Table S6: Spatial ANOVA test results of the watershed metals; Table S7: Sediment and riparian soil factor analysis result; Table S8. Soil and sediment PCA rotated component matrix; Table S9: Sediment risk assessment values; Table S10. Soil risk assessment values; Table S11: Seasonal metal risk assessment results in the watershed.
