# Supplementary material for: Riparian Soil Pollution Caused by Sediment Metal Transport: Seasonal Changes and Ecological Risk Assessment
Source: Toxics. 2024 Mar 13;12(3):213. doi: 10.3390/toxics12030213 (PMC10974470; doi:10.3390/toxics12030213)
Supplement: Supplementary file 1 [file toxics-12-00213-s001.zip › toxics-2869558-supplementary.pdf]

SUPPLEMENTARY MATERIAL

Table S1. Seasonal and spatial average metal concentration (mg/kg) of the sediment.

		Ba	Cr	Co	Cu	Fe	Pb	Mn	Ni	P	V	Zn	Al
Seasonal	Autumn	80.3	57.3	14.0	29.2	32792	5.111	636.6	50.9	605.7	78.0	41.9	21090
	Winter	68.5	43.0	14.5	30.7	34478	6.622	536.7	47.9	518.8	65.1	46.5	25659
	Spring	69.6	48.6	14.6	35.4	35256	6.244	596.3	50.3	440.6	69.0	42.7	25590
	Summer	61.3	42.5	14.3	27.9	32056	6.378	559.9	46.7	469.4	59.1	40.8	24599

Table S2. Seasonal and spatial average metal concentration (mg/kg) of the soil.

	Soil	Ba	Cr	Co	Cu	Fe	Pb	Mn	Ni	P	V	Zn	Al
Seasonal	Autumn	106.3	60.5	14.7	31.2	33793	7.71	619.6	49.9	673.2	80.2	51.9	23802
	Winter	92.5	44.1	15.5	30.2	34128	7.82	542.2	47.3	602.4	72.1	58.3	24380
	Spring	95.2	50.6	15.3	33.4	34546	6.66	578.2	50.1	582.5	80.3	54.4	24150
	Summer	88.3	46.3	14.7	29.7	32659	8.14	546.8	45.8	614.4	61.8	49.6	26422

Table S3. Sediment metal correlation in the watershed.

	Ba	Cr	Co	Cu	Fe	Pb	Mn	Ni	P	V	Zn	Al
Ba	1	-.515**	-.544**	-0.024	-.347*	.405*	.414*	-.597**	.626**	-0.197	0.014	.388*
Cr		1	0.212	-0.027	-.332*	-0.286	-.352*	.738**	-.329*	-0.165	-.389*	-.750**
Co			1	.347*	.332*	-.484**	-0.205	0.113	-.342*	0.310	-0.075	0.089
Cu				1	0.170	-0.266	-.415*	-0.152	-0.089	.381*	0.170	0.312
Fe					1	-0.236	-.331*	-0.312	-0.089	.838**	0.283	0.160
Pb						1	0.078	0.098	0.200	-.430**	0.296	0.123
Mn							1	-0.263	0.239	-.394*	-0.077	.413*
Ni								1	-.508**	-.398*	-0.236	-.564**
P									1	0.085	0.233	0.217
V										1	0.182	0.070
Zn											1	0.209
Al												1

Table S4. Riparian soil metal correlation in the watershed.

	Ba	Cr	Co	Cu	Fe	Pb	Mn	Ni	P	V	Zn	Al
Ba	1	-.933**	-.683*	0.133	0.300	-0.133	.833**	-.883**	-0.283	0.200	-0.317	0.483
Cr		1	0.550	-0.333	-0.483	0.033	-.717*	.933**	0.167	-0.367	0.000	-0.483
Co			1	0.317	-0.017	-0.267	-.683*	0.533	0.217	-0.050	0.383	-0.150
Cu				1	.700*	0.233	-0.133	-0.217	0.583	0.600	0.550	-0.150
Fe					1	0.350	-0.167	-0.583	0.317	.967**	0.617	-0.317
Pb						1	-0.233	0.050	0.400	0.367	0.417	-.717*
Mn							1	-0.550	-0.183	-0.267	-0.433	.700*
Ni								1	0.300	-0.517	0.000	-0.333
P									1	0.333	0.617	-0.300
V										1	0.600	-0.400
Zn											1	-0.267
Al												1

Table S5. Seasonal ANOVA test results of the watershed metals.

		ANOVA				
		Sum of Squares	df	Mean Square	F	Sig.
Ba	Between Groups	0.171	3	0.057	0.303	0.823
	Within Groups	6.018	32	0.188		
	Total	6.189	35			
Cr	Between Groups	0.496	3	0.165	0.604	0.617
	Within Groups	8.769	32	0.274		
	Total	9.266	35			
Co	Between Groups	0.046	3	0.015	0.524	0.669
	Within Groups	0.932	32	0.029		
	Total	0.978	35			
Cu	Between Groups	0.182	3	0.061	0.728	0.543
	Within Groups	2.661	32	0.083		
	Total	2.843	35			
Fe	Between Groups	0.042	3	0.014	0.378	0.769
	Within Groups	1.174	32	0.037		
	Total	1.216	35			
Pb	Between Groups	0.590	3	0.197	2.936	0.048
	Within Groups	2.142	32	0.067		
	Total	2.732	35			
Mn	Between Groups	0.059	3	0.020	0.220	0.882
	Within Groups	2.850	32	0.089		
	Total	2.909	35			
Ni	Between Groups	0.053	3	0.018	0.039	0.989
	Within Groups	14.439	32	0.451		
	Total	14.492	35			
P	Between Groups	0.377	3	0.126	3.837	0.019
	Within Groups	1.049	32	0.033		
	Total	1.427	35			
V	Between Groups	0.282	3	0.094	1.129	0.352
	Within Groups	2.660	32	0.083		
	Total	2.942	35			
Zn	Between Groups	0.057	3	0.019	0.896	0.454
	Within Groups	0.673	32	0.021		
	Total	0.729	35			
Al	Between Groups	0.347	3	0.116	1.718	0.183
	Within Groups	2.153	32	0.067		
	Total	2.500	35			

Table S6. Spatial ANOVA test results of the watershed metals.

		ANOVA				
		Sum of Squares	df	Mean Square	F	Sig.
Ba	Between Groups	4.616	8	0.577	9.908	0.000
	Within Groups	1.572	27	0.058		
	Total	6.189	35			
Cr	Between Groups	7.877	8	0.985	19.139	0.000
	Within Groups	1.389	27	0.051		
	Total	9.266	35			
Co	Between Groups	0.462	8	0.058	3.028	0.015
	Within Groups	0.515	27	0.019		
	Total	0.978	35			
Cu	Between Groups	1.552	8	0.194	4.056	0.003
	Within Groups	1.291	27	0.048		
	Total	2.843	35			
Fe	Between Groups	0.888	8	0.111	9.131	0.000
	Within Groups	0.328	27	0.012		

	Total	1.216	35			
Pb	Between Groups	1.576	8	0.197	4.600	0.001
	Within Groups	1.156	27	0.043		
	Total	2.732	35			
Mn	Between Groups	2.355	8	0.294	14.328	0.000
	Within Groups	0.555	27	0.021		
	Total	2.909	35			
Ni	Between Groups	13.546	8	1.693	48.308	0.000
	Within Groups	0.946	27	0.035		
	Total	14.492	35			
P	Between Groups	0.705	8	0.088	3.299	0.009
	Within Groups	0.721	27	0.027		
	Total	1.427	35			
V	Between Groups	2.139	8	0.267	8.988	0.000
	Within Groups	0.803	27	0.030		
	Total	2.942	35			
Zn	Between Groups	0.301	8	0.038	2.371	0.045
	Within Groups	0.428	27	0.016		
	Total	0.729	35			
Al	Between Groups	1.533	8	0.192	5.350	0.000
	Within Groups	0.967	27	0.036		
	Total	2.500	35			

Table S7. Sediment and riparian soil factor analysis result.

	Component	Initial Eigenvalues			Rotation Sums of Squared Loadings		
		Total	% of Variance	Cumulative %	Total	% of Variance	Cumulative %
Sediment	1	4.802	40.017	40.017	4.393	36.609	36.609
	2	3.923	32.695	72.711	3.928	32.736	69.345
	3	2.314	19.285	91.997	2.718	22.652	91.997
Soil	1	5.216	43.463	43.463	4.699	39.157	39.157
	2	3.486	29.054	72.517	3.768	31.401	70.559
	3	1.423	11.856	84.373	1.658	13.815	84.373

Table S8. Soil and sediment PCA rotated component matrix.

	Soil			Sediment		
	Component			Component		
	1	2	3	1	2	3
Ba	-0.050	**-0.961**	-0.117	-0.607	**0.643**	-0.452
Cr	0.046	**0.963**	-0.139	0.072	**-0.963**	-0.163
Co	**0.835**	0.421	-0.142	**0.820**	-0.459	0.205
Cu	**0.880**	-0.104	-0.244	**0.632**	-0.067	0.740
Fe	**0.934**	-0.145	0.181	**0.832**	0.449	0.209
Pb	0.102	-0.043	**0.966**	-0.087	0.156	**-0.972**
Mn	**-0.843**	-0.479	-0.091	**-0.818**	0.232	0.186
Ni	-0.083	**0.946**	-0.194	0.073	**-0.953**	-0.127
P	**0.641**	0.311	0.228	0.264	**0.846**	-0.290
V	**0.823**	-0.239	0.347	**0.768**	0.508	0.297
Zn	**0.705**	0.007	0.441	**0.930**	0.065	-0.081
Al	-0.197	**-0.655**	-0.409	-0.221	0.455	**0.824**

Extraction Method: Principal Component Analysis.

Rotation Method: Varimax with Kaiser Normalization.

Table S9. Sediment risk assessment values.

		Point										
		**1**	**2**	**3**	**4**	**5**	**6**	**7**	**8**	**9**	**PI**	**MPI**
CF	Cr	0.36	0.34	0.15	0.27	0.26	0.34	1.12	0.88	0.95	0.91	4.15
	Co	0.70	0.78	0.48	0.65	0.63	1.06	1.14	0.95	1.08	1.03	4.44
	Cu	0.97	0.85	0.48	0.95	1.13	1.95	1.25	1.04	1.29	1.58	5.25
	Pb	0.44	0.30	0.23	0.38	0.37	0.24	0.42	0.37	0.47	0.46	2.03
	Ni	1.45	0.90	0.61	1.05	0.88	1.15	5.32	4.13	4.80	4.29	19.24
	V	0.56	0.50	0.38	0.66	0.73	1.30	0.84	0.63	0.69	1.04	3.95
	Zn	0.62	0.51	0.41	0.58	0.68	0.75	0.73	0.67	0.82	0.79	3.39
	Al	0.31	0.26	0.15	0.20	0.21	0.37	0.22	0.21	0.25	0.32	3.99
EF	Cr	1.17	1.44	1.04	1.37	1.34	1.01	5.07	4.46	3.73		
	Co	2.27	3.07	3.13	3.29	3.14	2.92	5.11	4.74	4.24		
	Cu	3.14	3.41	3.17	4.72	5.37	5.29	5.57	5.07	5.06		
	Pb	1.42	1.18	1.49	1.87	1.85	0.66	1.90	1.79	1.84		
	Ni	4.64	3.58	4.03	5.21	4.34	3.43	23.99	20.72	18.84		
	V	1.83	2.02	2.56	3.41	3.89	3.59	3.78	3.20	2.72		
	Zn	1.98	2.01	2.69	2.92	3.38	2.07	3.25	3.30	3.23		
Igeo	Cr	-2.11	-2.28	-3.31	-2.48	-2.56	-2.17	-0.45	-0.77	-0.69		
	Co	-1.11	-0.99	-1.66	-1.21	-1.26	-0.51	-0.40	-0.67	-0.49		
	Cu	-0.68	-0.87	-1.67	-0.68	-0.48	0.33	-0.27	-0.55	-0.24		
	Pb	-1.81	-2.31	-2.73	-2.05	-2.08	-2.72	-1.84	-2.06	-1.70		
	Ni	-0.14	-0.74	-1.31	-0.55	-0.80	-0.46	1.81	1.45	1.66		
	V	-1.43	-1.60	-1.98	-1.19	-1.07	-0.22	-0.84	-1.27	-1.13		
	Zn	-1.33	-1.56	-1.89	-1.38	-1.22	-1.01	-1.05	-1.17	-0.89		
	Al	-2.29	-2.55	-3.30	-2.92	-2.88	-2.05	-2.75	-2.89	-2.57		
RI		29.8	25.3	16.6	27.5	25.9	41.4	60.0	48.1	57.1		
MRI		95.9	101.0	110.0	138.3	130.4	117.8	269.9	240.4	224.1		

Table S10. Soil risk assessment values.

		Point										
		1	2	3	4	5	6	7	8	9	PI	MPI
CF	Al	0.34	0.20	0.18	0.21	0.25	0.18	0.27	0.23	0.30	0.34	
	Co	0.78	0.74	0.68	0.74	0.82	0.86	1.15	0.82	1.05	1.17	4.91
	Cr	0.58	0.37	0.42	0.37	0.42	1.08	1.13	0.81	0.74	1.03	5.01
	Cu	1.06	0.99	0.85	1.10	1.28	1.03	1.56	0.92	1.48	1.59	6.38
	Fe	0.60	0.54	0.57	0.69	0.69	0.62	0.82	0.59	0.75	0.87	3.69
	Mn	5.31	1.28	1.22	1.48	1.77	1.35	1.50	1.36	2.09	4.22	13.58
	Ni	2.31	1.24	1.26	1.04	1.26	3.42	5.11	2.03	3.04	4.28	16.65
	Pb	0.49	0.31	0.73	0.36	0.46	0.42	0.51	0.30	0.50	0.69	3.57
	V	0.77	0.63	0.77	0.95	0.91	0.75	1.05	0.75	0.87	1.11	4.85

	Zn	0.81	0.62	0.73	0.65	0.84	0.66	1.04	0.71	0.92	1.07	4.44
EF	Co	2.32	3.61	3.90	3.59	3.23	4.67	4.30	3.62	3.53		
	Cr	1.74	1.81	2.39	1.80	1.66	5.83	4.23	3.61	2.49		
	Cu	3.17	4.86	4.84	5.37	5.03	5.58	5.85	4.08	4.98		
	Fe	1.80	2.62	3.26	3.38	2.70	3.33	3.07	2.60	2.51		
	Mn	15.84	6.28	6.98	7.23	6.93	7.28	5.63	6.03	7.03		
	Ni	6.89	6.07	7.19	5.08	4.94	18.48	19.17	9.00	10.20		
	Pb	1.46	1.50	4.19	1.76	1.79	2.27	1.91	1.35	1.69		
	V	2.30	3.09	4.41	4.62	3.56	4.05	3.92	3.33	2.92		
	Zn	2.41	3.01	4.18	3.16	3.30	3.59	3.88	3.16	3.09		
Igeo	Al	-2.16	-2.88	-3.10	-2.87	-2.56	-3.02	-2.49	-2.73	-2.33		
	Co	-0.94	-1.02	-1.13	-1.03	-0.87	-0.80	-0.39	-0.88	-0.51		
	Cr	-1.36	-2.02	-1.84	-2.03	-1.83	-0.48	-0.41	-0.88	-1.02		
	Cu	-0.50	-0.60	-0.82	-0.45	-0.23	-0.54	0.06	-0.71	-0.02		
	Fe	-1.31	-1.48	-1.39	-1.12	-1.12	-1.29	-0.87	-1.35	-1.01		
	Mn	1.82	-0.23	-0.30	-0.02	0.24	-0.16	0.00	-0.14	0.48		
	Ni	0.62	-0.28	-0.25	-0.53	-0.25	1.19	1.77	0.43	1.02		
	Pb	-1.61	-2.30	-1.03	-2.06	-1.71	-1.84	-1.56	-2.30	-1.57		
	V	-0.96	-1.25	-0.96	-0.66	-0.73	-1.00	-0.52	-1.00	-0.79		
	Zn	-0.89	-1.28	-1.04	-1.21	-0.83	-1.18	-0.53	-1.07	-0.70		
RI		31.36	22.12	24.30	23.36	26.57	38.62	55.27	28.46	40.17		
MRI		93.55	108.33	138.76	113.92	104.27	208.94	207.31	126.26	134.88		

Table S11. Seasonal metal risk assessment results in the watershed.

**autumn**		1	2	3	4	5	6	7	8	9	PI	MPI
CF	Cr	0.49	0.56	0.22	0.25	0.28	0.43	1.13	1.00	1.25	0.99	4.14
	Co	0.63	0.72	0.47	0.53	0.58	0.94	1.21	0.95	1.24	1.05	4.41
	Cu	1.00	0.90	0.56	0.69	0.74	1.57	1.49	1.04	1.42	1.33	5.48
	Pb	0.48	0.26	0.21	0.20	0.32	0.12	0.38	0.31	0.42	0.40	1.97
	Ni	1.72	0.94	0.65	0.70	0.72	1.26	5.31	3.89	5.91	4.49	17.17
	V	0.61	0.60	0.47	0.69	1.00	1.37	0.92	0.72	0.86	1.12	5.96
	Zn	0.56	0.52	0.45	0.46	0.53	0.68	0.87	0.71	0.86	0.76	3.52
	Al	0.29	0.19	0.13	0.14	0.14	0.30	0.25	0.20	0.27	0.26	
EF	Cr	1.70	2.96	1.64	1.79	2.03	1.40	4.53	5.11	4.65		
	Co	2.17	3.79	3.58	3.81	4.25	3.10	4.86	4.86	4.62		
	Cu	3.45	4.72	4.29	4.90	5.49	5.16	5.96	5.29	5.27		

	Pb	1.64	1.39	1.61	1.43	2.35	0.41	1.51	1.59	1.57
	Ni	5.95	4.95	4.91	5.01	5.31	4.16	21.30	19.86	21.96
	V	2.12	3.15	3.61	4.92	7.41	4.50	3.70	3.66	3.18
	Zn	1.93	2.74	3.40	3.27	3.90	2.25	3.50	3.64	3.18
Igeo	Cr	-1.61	-1.42	-2.79	-2.58	-2.45	-1.82	-0.41	-0.58	-0.26
	Co	-1.25	-1.06	-1.67	-1.49	-1.38	-0.67	-0.31	-0.66	-0.27
	Cu	-0.58	-0.74	-1.41	-1.13	-1.01	0.06	-0.01	-0.53	-0.08
	Pb	-1.65	-2.50	-2.82	-2.91	-2.24	-3.60	-1.99	-2.27	-1.82
	Ni	0.20	-0.67	-1.22	-1.10	-1.06	-0.25	1.82	1.37	1.98
	V	-1.29	-1.33	-1.66	-1.13	-0.58	-0.14	-0.70	-1.07	-0.81
	Zn	-1.43	-1.53	-1.74	-1.72	-1.51	-1.13	-0.78	-1.07	-0.81
	Al	-2.37	-2.98	-3.51	-3.42	-3.47	-2.30	-2.59	-2.94	-2.48
RI		31.44	27.47	17.82	22.61	24.46	38.16	60.89	47.22	66.18
MRI		108.4	144.7	135.4	161.6	180.6	125.67	244.36	241.12	245.98

**winter**		1	2	3	4	5	6	7	8	9	PI	MPI
CF	Cr	0.30	0.19	0.12	0.27	0.34	0.26	0.86	0.89	0.97	0.76	3.46
	Co	0.73	0.60	0.48	0.68	0.67	1.21	1.06	0.99	1.13	1.04	4.27
	Cu	0.89	0.53	0.41	1.04	1.22	1.97	1.26	1.02	1.53	1.59	4.99
	Pb	0.28	0.34	0.22	0.42	0.56	0.31	0.44	0.34	0.59	0.50	2.15
	Ni	0.76	1.03	0.55	1.12	1.18	0.73	4.50	4.88	5.12	3.94	18.71
	V	0.53	0.43	0.35	0.60	0.66	1.47	0.77	0.56	0.66	1.15	3.04
	Zn	0.43	0.53	0.41	0.64	1.09	0.86	0.67	0.61	1.01	0.91	4.03
	Al	0.28	0.31	0.16	0.20	0.22	0.46	0.23	0.20	0.27	0.37	
EF	Cr	1.10	0.61	0.78	1.35	1.56	0.57	3.82	4.48	3.53		
	Co	2.64	1.94	3.05	3.36	3.05	2.66	4.72	5.00	4.14		
	Cu	3.21	1.72	2.57	5.17	5.53	4.31	5.58	5.16	5.59		
	Pb	1.00	1.11	1.41	2.08	2.57	0.67	1.96	1.72	2.17		
	Ni	2.77	3.34	3.46	5.57	5.36	1.59	19.98	24.69	18.69		
	V	1.92	1.41	2.24	2.97	2.99	3.23	3.42	2.82	2.42		
	Zn	1.55	1.73	2.56	3.21	4.94	1.88	2.98	3.09	3.69		
Igeo	Cr	-2.31	-2.99	-3.60	-2.47	-2.13	-2.54	-0.80	-0.76	-0.63		
	Co	-1.04	-1.33	-1.63	-1.15	-1.16	-0.31	-0.50	-0.60	-0.40		
	Cu	-0.76	-1.50	-1.88	-0.53	-0.30	0.39	-0.25	-0.55	0.03		
	Pb	-2.44	-2.14	-2.75	-1.84	-1.41	-2.29	-1.77	-2.14	-1.34		
	Ni	-0.97	-0.55	-1.45	-0.42	-0.35	-1.04	1.58	1.70	1.77		
	V	-1.50	-1.79	-2.08	-1.33	-1.19	-0.03	-0.96	-1.42	-1.18		
	Zn	-1.81	-1.50	-1.89	-1.22	-0.46	-0.80	-1.16	-1.30	-0.57		
	Al	-2.44	-2.29	-3.24	-2.90	-2.77	-1.71	-2.74	-2.92	-2.45		
RI		22.08	25.57	14.64	26.50	31.27	38.30	54.03	50.72	62.73		
MRI		80.09	83.12	92.39	131.7	142.0	83.80	240.0	256.3	229.2		

**spring**		1	2	3	4	5	6	7	8	9	PI	MPI
CF	Cr	0.38	0.35	0.13	0.27	0.19	0.26	1.48	0.82	0.88	1.11	5.32
	Co	0.75	0.69	0.45	0.75	0.63	1.18	1.21	0.88	1.03	1.04	4.80
	Cu	1.37	0.90	0.44	1.11	1.63	2.74	1.11	0.81	1.26	2.13	5.77
	Pb	0.59	0.29	0.23	0.39	0.26	0.26	0.48	0.33	0.47	0.49	1.95
	Ni	2.05	0.77	0.53	1.09	0.76	0.89	6.45	3.73	4.59	4.85	23.23
	V	0.54	0.48	0.37	0.83	0.64	1.35	0.90	0.68	0.64	1.08	3.68
	Zn	0.90	0.50	0.39	0.60	0.44	0.84	0.69	0.56	0.81	0.78	3.14
	Al	0.35	0.27	0.17	0.26	0.25	0.41	0.21	0.16	0.25	0.34	
EF	Cr	1.10	1.29	0.76	1.05	0.75	0.63	7.13	5.29	3.57		
	Co	2.17	2.59	2.70	2.90	2.55	2.86	5.83	5.64	4.17		
	Cu	3.95	3.37	2.68	4.30	6.57	6.60	5.36	5.18	5.12		
	Pb	1.72	1.07	1.39	1.52	1.05	0.64	2.30	2.11	1.91		

	Ni	5.91	2.89	3.18	4.21	3.06	2.16	31.11	23.96	18.57
	V	1.55	1.79	2.22	3.18	2.58	3.26	4.34	4.33	2.57
	Zn	2.59	1.86	2.37	2.30	1.78	2.03	3.33	3.62	3.29
Igeo	Cr	-1.98	-2.12	-3.57	-2.46	-3.01	-2.52	-0.02	-0.86	-0.77
	Co	-1.00	-1.11	-1.75	-1.00	-1.25	-0.34	-0.31	-0.77	-0.54
	Cu	-0.13	-0.73	-1.76	-0.43	0.12	0.87	-0.43	-0.89	-0.25
	Pb	-1.34	-2.38	-2.71	-1.93	-2.53	-2.50	-1.65	-2.19	-1.67
	Ni	0.45	-0.95	-1.51	-0.46	-0.99	-0.75	2.10	1.32	1.61
	V	-1.48	-1.64	-2.04	-0.86	-1.24	-0.15	-0.74	-1.15	-1.24
	Zn	-0.74	-1.59	-1.94	-1.33	-1.77	-0.83	-1.12	-1.41	-0.88
	Al	-2.12	-2.48	-3.18	-2.53	-2.60	-1.86	-2.85	-3.27	-2.60
RI		39.05	21.71	15.67	30.67	24.89	39.62	70.10	44.21	54.96
MRI		112.8	80.93	94.96	118.2	100.7	95.57	338.0	283.7	222.6

**summer**		1	2	3	4	5	6	7	8	9	PI	MPI
CF	Cr	0.26	0.25	0.15	0.28	0.24	0.43	1.03	0.82	0.71	0.80	3.66
	Co	0.67	1.10	0.50	0.66	0.64	0.92	1.07	0.97	0.90	0.97	4.28
	Cu	0.63	1.07	0.49	0.96	0.91	1.51	1.15	1.30	0.95	1.28	4.78
	Pb	0.42	0.32	0.24	0.52	0.34	0.27	0.39	0.48	0.38	0.46	2.06
	Ni	1.27	0.88	0.72	1.29	0.85	1.73	5.02	4.01	3.61	3.86	17.83
	V	0.55	0.48	0.34	0.55	0.61	1.00	0.78	0.55	0.60	0.83	3.15
	Zn	0.59	0.48	0.38	0.62	0.68	0.63	0.68	0.79	0.62	0.71	2.89
	Al	0.32	0.28	0.16	0.21	0.24	0.30	0.21	0.28	0.22	0.29	
EF	Cr	0.79	0.89	0.98	1.31	1.02	1.44	4.81	2.95	3.18		
	Co	2.07	3.97	3.21	3.09	2.70	3.09	5.01	3.46	4.01		
	Cu	1.96	3.84	3.12	4.52	3.88	5.07	5.39	4.66	4.25		
	Pb	1.31	1.14	1.54	2.45	1.45	0.91	1.85	1.73	1.71		
	Ni	3.92	3.15	4.58	6.07	3.62	5.80	23.55	14.39	16.15		
	V	1.71	1.73	2.18	2.56	2.60	3.36	3.68	1.99	2.70		
	Zn	1.84	1.73	2.44	2.91	2.90	2.10	3.21	2.85	2.77		
Igeo	Cr	-2.55	-2.60	-3.29	-2.42	-2.65	-1.80	-0.55	-0.87	-1.08		
	Co	-1.16	-0.44	-1.57	-1.19	-1.24	-0.71	-0.49	-0.64	-0.74		
	Cu	-1.25	-0.49	-1.62	-0.64	-0.71	0.01	-0.38	-0.21	-0.66		
	Pb	-1.82	-2.24	-2.64	-1.52	-2.14	-2.47	-1.93	-1.64	-1.97		
	Ni	-0.24	-0.78	-1.06	-0.21	-0.82	0.20	1.74	1.42	1.27		
	V	-1.44	-1.64	-2.13	-1.46	-1.29	-0.58	-0.94	-1.44	-1.31		
	Zn	-1.34	-1.64	-1.97	-1.27	-1.14	-1.26	-1.13	-0.92	-1.28		
	Al	-2.21	-2.43	-3.26	-2.81	-2.67	-2.33	-2.81	-2.43	-2.75		
RI		26.59	26.46	18.36	30.25	23.13	49.49	54.88	50.37	44.41		
MRI		82.23	95.05	117.0	141.6	98.27	166.1	257.3	180.5	198.8		
